# The Prevalence of Second Neoplasms in Patients with Non-Aldosterone Producing Adrenocortical Lesions

**DOI:** 10.3390/ijms262010167

**Published:** 2025-10-19

**Authors:** Paraskevi Tripolitsioti, Ariadni Spyroglou, Odysseas Violetis, Panagiota Konstantakou, Eleni Chouliara, Grigoria Betsi, Konstantinos Iliakopoulos, Eleni Memi, Konstantinos Bramis, Denise Kolomodi, Paraskevi Xekouki, Manousos Konstadoulakis, George Mastorakos, Krystallenia I Alexandraki

**Affiliations:** 1 Intensive Care Unit, 1st Department of Respiratory Medicine, National and Kapodistrian University of Athens, Sotiria Hospital, 11527 Athens, Greece; par.tripolitsioti@gmail.com; 2 2nd Department of Surgery, Aretaieio Athens Hospital, Medical School, National and Kapodistrian University of Athens, 11528 Athens, Greece; aspyroglou@yahoo.com (A.S.); odysseas.violetis@gmail.com (O.V.); panagiotaki05@hotmail.com (P.K.); lenaxou1995@gmail.com (E.C.); kiliakopoulos1@gmail.com (K.I.); eleni.memi@gmail.com (E.M.); kbramis@gmail.com (K.B.); mkonstad@med.uoa.gr (M.K.); gmastorak@med.uoa.gr (G.M.); 3 Endocrinology & Diabetes Clinic, University Hospital of Heraklion, University of Crete, School of Medicine, 71500 Crete, Greece; raniabetsi1979@gmail.com (G.B.); pxekouli@uoc.gr (P.X.); 4 European Neuroendocrine Tumor Society (ENETS) Center of Excellence, Ekpa-Laiko Center, 11527 Athens, Greece; denisekol.dk@gmail.com; 5 IATROPOLIS Private Medical Center, 11521 Athens, Greece; 6 Unit of Endocrinology, Diabetes Mellitus and Metabolism, Aretaieion Hospital, Faculty of Medicine, National and Kapodistrian University of Athens, 11528 Athens, Greece

**Keywords:** non-aldosterone producing adrenal adenomas (NAPACA), second neoplasm, second malignancy

## Abstract

Over the last few decades, due to improvement in imaging techniques, the increased detection of adrenal incidentalomas is observed. Non-aldosterone producing adrenal adenomas (NAPACAs) often co-exist with second benign or malignant lesions. In the present study, we aimed to assess the presence of second neoplasms, both benign and malignant, in patients with NAPACAs, and to investigate possible correlations with clinical parameters, hormonal characteristics and the emergence of comorbidities. A total of 130 NAPACA patients were included in this single-center retrospective study. In this cohort, 35.4% of NAPACA patients carried any second neoplasm (either benign or malignant) whereas, 26.9% had a second malignant neoplasm. Cortisol levels after 1 mg overnight dexamethasone suppression test (F-ODS) were significantly higher in patients without a second neoplasm (*p* = 0.02), and this finding was consistent even when categorizing patients with and without malignancies (*p* = 0.02). In line with this observation, ACTH/F-ODS levels were significantly higher in patients with second malignancies (*p* < 0.05). Interestingly, the presence of mild autonomous cortisol secretion tended to be lower in patients with second malignancies (*p* = 0.08). No remarkable differences in the comorbidities of NAPACA patients with and without a second neoplasm were documented. Further prospective studies will be needed to elucidate the role of mild hypercortisolemia on the development of these second tumors in NAPACA patients.

## 1. Introduction

Due to the progressively wider availability and applicability of cross-sectional imaging techniques, an increased incidental detection of clinically silent adrenal masses is observed. The prevalence of adrenal incidentalomas rises with age and ranges between 5 and 10% in recent studies [1,2,3,4,5]. Non-aldosterone producing adrenal adenomas (NAPACAs) are benign adrenal adenomas, usually identified as incidentalomas, without hormonal hypersecretion. However, a detailed work-up might reveal biochemical evidence of mild hypercortisolism in patients with NAPACAs. This mild autonomous cortisol secretion (MACS) correlates with increased morbidity, in particular, cardiovascular comorbidities, such as type 2 diabetes mellitus and dyslipidemia [6,7]. Interestingly, the prevalence of cortisol-secreting adrenal incidentalomas reaches 11.7%, rendering this feature relevant for the surveillance of comorbidities [8]. Furthermore, the increased risk of metabolic diseases has been documented in NAPACA patients throughout a continuum from non-secreting NAPACAs to MACS [9,10].

The co-existence of multiple neoplasms, either benign or malignant, in the same patient is not a rare finding; however, so far, possible links between these neoplasms have only been investigated in the context of rather rare genetic syndromes. In the field of endocrine tumors, a previous nationwide Swedish study identified an increased risk for a second endocrine neoplasm in patients with a first endocrine tumor, with adrenal neoplasms presenting a standardized incidence ratio of 10 in combination with thyroid neoplasms [11]. In a smaller study, adrenal incidentaloma patients carrying a second malignancy presented similar clinical behavior of the adrenal tumor in comparison to patients without a second neoplasm but who were older and presented a larger tumor growth [12]. In a recent large study, one third of the patients with adrenal incidentaloma presented further adenomas in other organs, and the presence of mild hypercortisolism did not affect the emergence of second neoplasms [13]. The role of cortisol in the development of both benign and malignant neoplasms has been discussed so far with controversial findings; NAPACAs present a fertile field for further investigation of these observations [14,15,16,17].

The aim of the present study is to assess the presence of second neoplasms, both benign and malignant, in patients with NAPACA, and to investigate possible correlations with clinical parameters, hormonal characteristics, and the emergence of comorbidities in these patients both at the time of diagnosis as well as at the time of the latest follow-up.

## 2. Results

In our cohort, the presence of any type of second neoplasm was identified in 46/130 (35.4%) of the NAPACA patients, while 35/130 (26.9%) of the patients carried a second malignant neoplasm within a median follow-up time of four years (range 2–34 years). Three patients carried more than one malignancy, while five patients carried both malignant and benign neoplasms. The diagnosis of these neoplasms preceded the NAPACA diagnosis in 25 (64%) patients with malignant and in 10 (50%) patients with non-malignant second neoplasms, was synchronous with the NAPACA diagnosis in 7 (18%) patients with malignant and in 4 (20%) patients with non-malignant second neoplasms, and followed NAPACA diagnosis in 7 (18%) patients with malignant and in 6 (30%) patients with non-malignant second neoplasms. The detailed distribution of second benign and malignant neoplasms, together with their emergence prior (pre), simultaneously (syn), or after the diagnosis (meta) of NAPACA, can be found in Table 1.

Although the vast majority of our patients were women (78.5%), no significant gender differences were documented between NAPACA patients with and without any second neoplasm or malignancy (*p* = 0.626 and 0.482). Patients of the different subgroups (presence versus non-presence of any second neoplasm, presence versus non-presence of any second malignant neoplasm, respectively) did not differ in their age at diagnosis (*p* = 0.285 for any second neoplasm and *p* = 0.711 for second malignant neoplasm), bodymass-index (BMI), and maximum NAPACA size. Furthermore, no differences could be documented in the presence of comorbidities, such as hypertension, dyslipidemia, chronic kidney disease, osteoporosis, psychiatric disorders, and cardiovascular thrombosis (myocardial infraction—percutaneous transluminal coronary angioplasty (PTCA), stroke, deep vein thrombosis, and pulmonary embolism).

From the hormonal parameters investigated, cortisol levels after 1 mg overnight dexamethasone suppression test (F-ODS) were significantly higher in patients without a second neoplasm (with second neoplasm F-ODS: 1.0 (0.41–3.0) μg/dL; without second neoplasm F-ODS: 1.25 (0.30–4.33) μg/dL, *p* = 0.02), and this finding was consistent when categorizing patients with and without malignancies (with second malignant neoplasm F-ODS: 1.0 (0.41–3.0) μg/dL vs. without second malignant neoplasm F-ODS: 1.20 (0.30–4.33) μg/dL, *p* = 0.02). Interestingly, this difference did not retain statistical significance in the follow-up investigation. ACTH levels and the ratio DHEA-S to the lower limit of normal (DHEA-S-to-LLN) did not differ among the different groups at baseline, but ACTH levels tended to be lower in patients without any second neoplasm or second malignancy at follow-up (with any second neoplasm ACTH: 20.75 (5–97.9) pg/mL; without any second neoplasm ACTH:16.75 (4.52–65.3) pg/mL, *p* = 0.05; with second malignant neoplasm ACTH: 23.6 (5–97.9) pg/mL; and without second malignant neoplasm ACTH:17.1 (4.52–65.3) pg/mL, *p* < 0.05). The presence of MACS did not significantly affect the presence of any second neoplasm or second malignant neoplasm either at the baseline or follow-up. When calculating the ACTH to F-ODS ratio, NAPACA patients with second malignant neoplasm displayed significantly higher ratios both at the baseline visit and at follow-up (baseline: with second malignant neoplasm ACTH/F-ODS: 23.65 (2.5–214) vs. without second malignant neoplasm: 12.64 (0.92–222), *p* < 0.05; follow-up: with second malignant neoplasm ACTH/F-ODS: 23.38 (2.43–163) vs. without second malignant neoplasm: 12.18 (0.50–163), *p* < 0.05), indicating that the lower cortisol secretion, paired with slightly higher ACTH levels, correlates with the presence of second malignant neoplasms.

Although no significant differences were documented in the diabetes presence at baseline, the patients with any second neoplasm more frequently presented with diabetes at follow-up (37% vs. 19%, *p* < 0.05). Dementia was more frequent in patients with any second neoplasm (6.5% vs. 0%, *p* < 0.05) both at baseline and at follow-up and heart failure in patients with second malignancy at follow-up (5.7% vs. 0%, *p* < 0.05). Unlike that, smoking was more frequent in patients without any second neoplasm (48.8% vs. 27.9%, *p* < 0.05). When comparing baseline with the follow-up prevalence, metabolic comorbidities (hypertension, type 2 diabetes mellitus, and dyslipidemia) and osteoporosis presented a significant increase in their presence in all NAPACA patients and in the subgroups of patients without any second neoplasms or without second malignant neoplasms (Table 2).

To further investigate the effect of MACS in NAPACA patients with and without second neoplasms, we additionally compared the characteristics of patients with and without MACS at baseline and follow-up. As expected, MACS patients were older, displayed significantly higher F-ODS levels (*p* < 0.001) and lower ACTH levels (*p* < 0.001), and the presence of MACS was associated with a significantly increased frequency of comorbidities (hypertension, diabetes mellitus type 2, dyslipidemia, cardiovascular thrombosis, PTCA, atrial fibrillation, and psychiatric disorders). However, both the presence of any second neoplasms and second malignancies was more frequent in patients without MACS, still without reaching statistical significance (*p* = 0.089 and 0.058, respectively). Both groups of patients, with and without MACS, presented a significant worsening of their metabolic comorbidities (hypertension, type 2 diabetes, and dyslipidemia) between baseline and follow-up visits (Table 3).

All parameters were submitted to univariate regression analysis followed by a multivariate analysis (age, gender, BMI, F-ODS, ACTH, DHEA-S to LLN, BMI/F-ODS, ACTH/F-ODS, tumor size, delta tumor size, smoking, and time of follow-up). The univariate logistic regression analysis revealed that only ODS at baseline and the change in NAPACA size could predict the presence of any type of neoplasms and that ODS at baseline, the change in NAPACA size, and ACTH at the last follow-up could predict the presence of a malignant neoplasm (Table 4). Furthermore, we performed a Receiver Operating Characteristic analysis for ODS in the identification of second neoplasms and second malignancies and documented a sensitivity of 0.674 in the identification of any second neoplasms and of 0.629 in the identification of second malignancies (*p* = 0.021 and 0.021, respectively), with a proposed F-ODS cut-off of 1.06 μg/dL for any second neoplasms and 1.79 μg/dL for second malignancies.

## 3. Discussion

In the present study, we examined the clinical characteristics of 130 NAPACA patients followed-up for at least 24 months and found a prevalence of 35% for any second neoplasms and 27% for second malignant neoplasms in this cohort, with the majority of neoplasms preceding the appearance of NAPACA. This prevalence is in line with two previous studies that documented the presence of second neoplasms, benign in the first and malignant in the second, in approximately one third of patients with adrenal incidentalomas despite the different methodology used in these studies [12,13]. In accordance with the study by Tsvetov et al., we did not observe any unexpected aggregation of a particular or rare malignancy [12]. In the context of benign tumors, we observed an increased frequency of further endocrine adenomas/hyperplasias, analogous to the findings of Herrera et al., but the small total number of these neoplasms in our cohort does not allow for any safe conclusions. It is also of interest that, in our study, most second neoplasms precede the emergence of adrenal incidentaloma, in convergence with the study byTsvetov, which only included patients with preceding second malignancy. A possible explanation for this is the fact that patients with prior/concurrent malignancies undergo an intense surveillance in comparison to cancer-naïve patients, enabling the detection of further incidentalomas.

We additionally compared the demographic characteristics and comorbidities of patients with and without second neoplasms at baseline and at a median follow-up of four years. In our cohort, NAPACA diagnosis occurred at a median age of 61 years, with a female prevalence and a median BMI of 28.7 kg/m^2^, in accordance with previous studies [6,18] and without significant differences between patients with and without a second neoplasm. The presence of comorbidities in our cohort (hypertension, type 2 diabetes mellitus, and dyslipidemia) did not differ from previous studies [6]. Interestingly, although the frequencies of these comorbidities did not differ between patients with and without second neoplasms at baseline, the prevalence of diabetes was significantly higher in patients with any second neoplasms at follow-up, a finding that was not due to the higher F-ODS levels, suggesting that other factors possibly related to the second neoplasm and its specific treatment rather than to the high cortisol affect diabetes progression. On the other hand, as expected, when comparing the prevalence of these comorbidities between patients with or without MACS, MACS patients displayed a significantly higher prevalence of hypertension, diabetes, and dyslipidemia, as well as cardiovascular thrombotic events, in line with previous studies [2,6,9,19]. However, we did not document a significant progression of the majority of the comorbidities, besides hypertension, diabetes, and dyslipidemia, between baseline and the last follow-up. A possible explanation for this phenomenon could be the rather small number of patients and the respective time frame that did not allow relevant changes to occur in our cohort. The observed progression of hypertension, diabetes and dyslipidemia in this cohort can of course be attributed to an age-dependent deterioration of these conditions. The unexpected observation of a reduced prevalence of second neoplasm in smokers can be attributed to the fact that only current smoking was recorded in our study and that patients with a diagnosis of a neoplasm probably change their habits and quit smoking more frequently.

We further investigated the hormonal characteristics of NAPACA patients, both at baseline and at follow-up, aiming to investigate possible links between these and the presence of second neoplasms in our population. Remarkably, both patients with any second neoplasm and patients with second malignant neoplasms displayed significantly lower F-ODS values at baseline. In addition to this, the ACTH/F-ODS ratio was significantly higher in patients with second malignant neoplasms. Although the presence of MACS in these patients only tended to decrease but was not significantly lower than in patients without neoplasm, all these findings are suggestive of a possibly protective role of mild hypercortisolemia in the development of second neoplasms. The lack of significant MACS differences could be explained by the presence of a continuum, where only mildly elevated F-ODS values, still within the normal range, exert this role. In order to strengthen this finding, we compared the prevalence of patients with and without MACS and we found a tendencyofstatistically lower prevalences of neoplasms in patients with NAPACAs and MACS. On top of that, the regression analysis highlighted the F-ODS as the sole factor assessed at the time of diagnosis, which is able to predict the presence of a second neoplasm, benign or malignant. Unlike this, Tsvetov et al., did not document any differences between patients with and without hypercortisolism, but Herrera et al. showed significantly higher 24 h urinary cortisol levels only in MACS patients without a second neoplasm [12,13]. These discrepancies may be due to the in part small cohorts and the differences in the inclusion criteria used. Whether chronic cortisol exposure exerts immunomodulatory effects other than immunosuppression, which is acknowledged to promote tumorigenesis, is unclear. Mild hypercortisolism couldmodulate the tumor microenvironment, and it is already observed that glucocorticoid receptor activation on specific hormone-dependent tumor entities (breast and prostate cancer) can inhibit tumor cell growth and proliferation [20,21]. On the other hand, a reverse causality should be discussedwhere, in particular, for pre-existing neoplasms, the stress response and cortisol secretion associated with the diagnosis or treatment of the malignancy could have influenced the emergence or detection of a NAPACA.

Although several previous studies in the literature deal with the presence of adrenal lesions in patients with other malignancies, these rarely approach the question of a possible link between adrenal adenoma and a second neoplasm but mainly focus on the differential diagnosis of newly diagnosed adrenal masses in oncological patients [22,23,24,25]. In particular, the question of second neoplasms has already been discussed in the context of neuroendocrine tumors [26,27]. However, the presence of a second neoplasm using as the starting point the presence of adrenal incidentalomas has been discussed in two studies. The study by Tsvetov et al. discusses 100 cases with second malignant neoplasms preceding the diagnosis of incidentalomas, and these, in turn, do not only include NAPACAs but also tumors related to secretory syndromes (primary aldosteronism, Cushing Syndrome, and pheochromocytoma) [12]. The second study focuses on second endocrine adenomas in patients with adrenal adenomas in 923 patients and also registers the metabolic and cardiovascular comorbidities in the different patient groups without a follow-up over time [13]. Unlike these previous studies, the present study design focuses only on NAPACAs, excluding secretory adenomas, covers the emergence of both second benign and malignant neoplasms preceding, synchronous to, and metachronous to diagnosis, and also provides follow-up data on the comorbidities of these patients, presenting a comprehensive characterization of this co-existence.

Even if this study characterizes, in depth, the traits of NAPACA patients with and without neoplasms and observes their evolution over time, it presents some limitations. The retrospective nature of the study comprises a risk of bias, and larger patient numbers would also strengthen the interpretation of the results. Taken together, we documented herein a lower prevalence of second neoplasms in NAPACA patients displaying higher cortisol levels, even if these cortisol levels were not necessarily classified as MACS. Additional large prospective studies will be needed to further investigate these findings on NAPACA and the co-existence of second neoplasms and to possibly substantiate the role of mild hypercortisolemia on the development of these second tumors.

## 4. Materials and Methods

**Figure 1:**
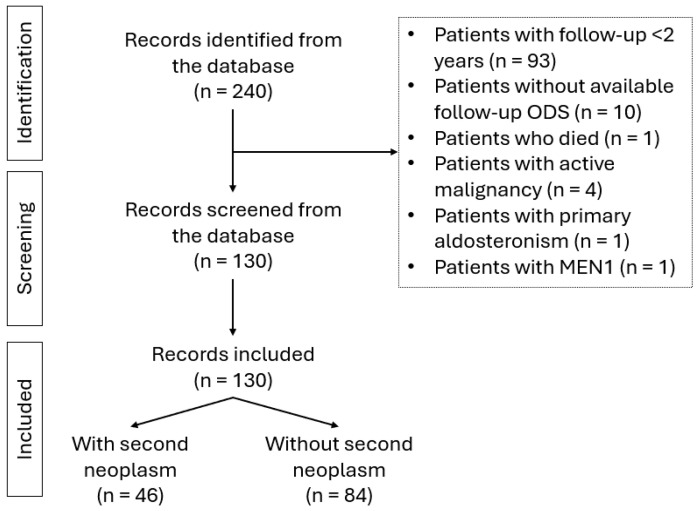
STROBE flow diagram for included patients.

This is a single-center retrospective study, performed at the 2nd Department of Surgery, Aretaieion hospital, Athens, Greece (Ethical Approval from the Ethics Committee of the Aretaieion Hospital, Decision Nr. 363/13-10-2021). Informed consent was obtained from all patients before study enrollment. The study is presented herein according to the STROBE reporting guidelines (Figure 1).

A total of 240 patients with NAPACA presented in the outpatient clinic between 2020 and 2025; 130 patients had follow-up data for at least 24 months. Patients <18 years old, with active malignancy, with genetic syndromes related to the presence of NAPACA, patients with hormonally active adenomas (overt Cushing Syndrome, pheochromocytoma, or primary aldosteronism) were excluded. According to the European Network for the Study of Adrenal Tumors (ENS@T) definition, NAPACA refers to all patients with non-aldosterone producing adrenocortical adenoma. That includes both patients with non-secretory adenomas (cortisol after overnight 1 mg dexamethasone suppression (F-ODS) < 1.8 μg/dL) but also patients with MACS (F-ODS < 5 μg/dL), according to the cut-offs provided by the European Society of Endocrinology clinical practice guidelines for the management of adrenal incidentalomas [4]. Patients with MACS do not present clinically overt Cushing Syndrome and were included in the present study. The benign nature of the adrenal lesion was warranted by radiological characteristics (homogenous, <10 HU in unenhanced CT, relative washout > 58% in CT with contrast media, loss of signal intensity on out-phase imaging in MRI, and absence of FDG uptake or uptake less than the liver in FDG-PET/CT) both at diagnosis and at 24 months of follow-up.

We have also included patients with NAPACAs less than 1 cm in diameter, with a minimum diameter of 8 mm, since the ESE guidelines explicitly state application of an arbitrary work-up threshold of 1 cm [4]. Data on the demographic characteristics of the patients, such as gender, age, weight (kg), height (cm), smoking habits, and NAPACA size (mm) and hormonal parameters, such as F-ODS (μg/dL), ACTH (pg/mL), and DHEA-S (μg/dL), measured by conventional assays, as well as the presence of comorbidities (hypertension, diabetes mellitus type 2, dyslipidemia, chronic kidney disease, osteoporosis, fractures, dementia, psychiatric disorders, cardiovascular thrombosis, myocardial infraction, PTCA, atrial fibrillation, stroke, deep venous thrombosis, and pulmonary embolism), were documented at baseline and at least at a 24-month follow-up visit. Due to age and gender dependent variations in the normal range of DHEA-S, we calculated the DHEA-S to the lower reference limit ratio (DHEA-S to LLN) and applied this to the analysis. Furthermore, the BMI was calculated according to the following formula, weight (kg)/(height in m^2^). Additionally, the ACTH/F-ODS and the BMI/F-ODS were calculated. The ACTH/F-ODS ratio calculation relies on the fact that physiologically, in NAPACAs, the increase in cortisol secretion correlates with an ACTH suppression. The difference in the NAPACA size between baseline and follow-up visit was also calculated (delta size, in mm). We further registered second neoplasms occurring in NAPACA patients and categorized patients as follows: “Without any second neoplasm” vs. “with second neoplasm” (here including both benign and/or malignant second tumors) or for a nested analysis “without second malignant neoplasm” vs. “with second malignant neoplasm”.

Continuous variables are presented as median value and range. Categorical variables are presented as percentage of the population studied. Correlations between categorical variables were assessed by the chi-square test. Especially, chi-square test was applied for the analysis of gender, smoking habit, hypertension, diabetes mellitus type 2, dyslipidemia, chronic kidney disease, osteoporosis, fractures, dementia, psychiatric disorders, cardiovascular thrombosis, myocardial infraction, PTCA, atrial fibrillation, stroke, deep vein thrombosis, presence of MACS, and bilateral localization of NAPACA. Mann–Whitney test was performed for continuous non-parametric variables (age, BMI, F-ODS, ACTH, DHEA-S to LLN, max. size of the NAPACA, BMI/F-ODS, and ACTH/F-ODS). Wilcoxon paired test was used to compare the continuous variables at baseline and last follow-up, whereas qualitative variables were compared with McNemar test. Odds ratio (OR) and 95% Confidence Interval (CI) for binary outcomes in univariate and multivariate logistic regression models were calculated and reported using the presence or absence of second neoplasms as dependent variables. The results were considered statistically significant when the *p*-value was <0.05. Analysis was performed using SPSS (version 30.0.0.0 (172); SPSS, Inc., Chicago, IL, USA) for Windows XP (Microsoft Corp., Washington, DC, USA).

## Figures and Tables

**Table 1:** Distribution (pre-existent, synchronous, or metachronous) of malignant and non-malignant second neoplasms in patients with NAPACA.

Malignant Neoplasms	N	Pre	Syn	Meta
Breast Carcinoma	6	4		2
Colorectal Carcinoma	6	5		1
Differentiated Thyroid Carcinoma	4	3	1	
Prostate Carcinoma	3	3		
Endometrial Carcinoma	3	1	2	
Pancreatic Neuroendocrine Tumor	3	2		1
Skin Carcinoma (Squamous or Basal Cell Carcinoma)	3		1	2
Gastric Neuroendocrine Neoplasm Type 1	2		1	1
Renal Carcinoma	2	1	1	
Lung Carcinoma	1	1		
Melanoma	2	2		
Bladder Carcinoma	1	1		
Pancreatic Carcinoma	1	1		
Appendiceal Neuroendocrine Neoplasm	1	1		
Medullary Thyroid Cancer	1		1	
TOTAL	39	25	7	7
**Benign Neoplasms**	**N**	**Pre**	**Syn**	**Meta**
ECL-cell Gastric Hyperplasia	3	2		1
Pituitary Adenoma	4	1	3	
Lipoma (Retroarticular Area/Cecum)	2	1		1
Parathyroid Adenoma	3	2		1
Parotid Neoplasm (Pleiomorphic Adenoma, Warthin’s Tumor)	2	2		
Breast Neoplasm (Phyllodes Tumor)	1	1		
Angiolipoma	1			1
Meningioma	1			1
Myeloma	1			1
IPMN	1		1	
Colon Polyp	1	1		
TOTAL	20	10	4	6

ECL: enterochromaffin-like cells, and IPMN: intraductal papillary mucinous neoplasm.

**Table 2:** Demographics, hormonal data, and comorbidities of patients with NAPACA, with and without any second neoplasms and with and without second malignant neoplasms at baseline (left columns) and at the latest follow-up visit (>24 months) (right columns). Respective parameters of patients with and without second neoplasms or with and without second malignancies were compared with χ^2^ test for categorical variables and with Mann–Whitney test for continuous variables. The respective *p*-values can be found in the columns on the right. For the comparison between the respective parameters at baseline vs. follow-up, the McNemar test was applied for categorical values and the Wilcoxon-paired test for continuous variables. The respective *p*-values are denoted with * in the respective median or frequency.

	BASELINE							FOLLOW-UP						
	All Patients N = 130	WITH 2nd Neoplasm N = 46	NO 2nd Neoplasm N = 84	*p*	WITH 2nd Malignant Neoplasm N = 35	NO 2nd Malignant Neoplasm N = 95	*p*	All Patients N = 130	WITH 2nd Neoplasm N = 46	NO 2nd Neoplasm N = 84	*p*	WITH 2nd Malignant Neoplasm N = 35	NO 2nd Malignant Neoplasm N = 95	*p*
**Age (years)**	61 (18–89)	63 (25–85)	61 (18–89)	*0.285*	61 (25–81)	61 (18–89)	*0.711*	67 (28–91)	68 (30–89)	65 (28–91)	*0.497*	67 (30–89)	66.5 (28–91)	*0.975*
**Male (n,%)**	28 (21.5%)	11 (23.9%)	17 (20.2%)	*0.626*	9 (25.7%)	19 (20.0%)	*0.482*	Not applicable						
**BMI (kg/m^2^)**	28.70 (17.14–63.29) **	28.19 (17.14–45.61) **	28.74 (18.03–63.29)	*0.445*	27.70 (17.14–45.61)	28.75 (18.03–63.29)	*0.537*	28.68 (17.40–55.08)	27.88 (17.40–44.50)	28.91 (17.63–55.08)	*0.234*	27.78 (17.40–44.50)	28.84 (17.63–55.08)	*0.299*
**F-ODS (μg/dL)**	1.15 (0.30–4.33) **	1.00 (0.41–3.00)	1.25 (0.30–4.33)	*0.02*	1.00 (0.41–3.00)	1.20 (0.30–4.33)	*0.02*	1.10 (0.30–10.70)	1.03 (0.49–4.9)	1.20 (0.30–10.70)	*0.087*	1.01 (0.49–4.9)	1.20 (0.30–10.70)	*0.082*
**ACTH (pg/mL)**	16.7 (3.8–200)	19.4 (5–107)	16.6 (3.8–200)	*0.302*	21.6 (5–107)	16.6 (3.8–200)	*0.297*	18.4 (4.52–97.9)	20.75 (5–97.9)	16.75 (4.52–65.3)	*0.056*	23.6 (5–97.9)	17.1 (4.52–65.3)	*0.031*
**DHEA-S to LLN**	2.18 (0.42–13.04)	2.15 (0.51–10.17)	2.20 (0.42–13.04)	*0.868*	2.15 (0.51–8.00)	2.18 (0.42–13.04)	*0.840*	2.54 (0.34–13.04)	2.6 (0.51–12.28)	2.53 (0.34–13.04)	*0253*	3.02 (0.51–12.28)	2.52 (0.34–13.04)	*0.339*
**MACS (n,%)**	33 (25.4%)	8 (17.4%)	25 (29.8%)	*0.121*	5 (14.3%)	28 (29.5%)	*0.078*	38 (29.2%)	7 (15.2%)	31 (36.9%)	*0.195*	11 (31.4%)	27 (28.4%)	*0.420*
**BMI/F-ODS (kg/m^2^/μg/dL)**	25.09 (6.27–120.17)	28.94 (6.84–68.75)	22.76 (6.27–120.17)	*0.069*	28.44 (8.45–68.75)	23.85 (6.27–120.17)	*0.094*	25.02 (2.68–128.09)	27.25 (6.13–69.53)	23.53 (2.68–128.09)	*0.148*	28.09 (7.60–69.53)	23.55 (2.68–128.09)	*0.101*
**ACTH/F-ODS (pg/mL/μg/dL)**	14.20 (0.92–222.22)	17.62 (2.50–214.00)	13.00 (0.92–222.22)	*0.256*	23.65 (2.50–214.00)	12.64 (0.92–222.22)	*0.046*	13.35 (0.50- 163.25)	23.04 (2.43–163.17)	12.42 (0.50–163.25)	*0.036*	23.38 (2.43–163.17)	12.18 (0.50–163.25)	*0.014*
**Tumor size (mm)**	8 (4.5–42) *	8 (4.5–35)	10 (5–42) *	*0.420*	8 (4.5–35)	8.5 (5–42) *	*0.769*	11 (4.5–48)	8.5 (4.5–35)	13 (4.7–48)	*0.244*	10 (4.5–35)	11.5 (4.7–48)	*0.492*
**Delta size (mm)**	Not applicable							0 (−8 to 25.5)	0 (−6 to 8)	0 (−8 to 25.5)	*0.129*	0 (−6 to 8)	0 (−8 to 25.5)	*0.03*
**Bilateral (n,%)**	44/129 (34.1)	16 (34.8)	28/83 (33.7)	*0.528*	13 (37.1)	31/94 (33.0)	*0.524*	51/129 (39.5)	18 (39.1)	33/83(39.8)	*0.548*	14 (40.0)	37/94 (39.4)	*0.508*
**Smokers (n,%)**	52/125 (41.6)	12/43 (27.9)	40/82 (48.8)	*0.024*	10/33 (30.3)	42/92 (45.7)	*0.125*	Not assessed						
**HTN (n,%)**	64 (49.2) *	24 (52.2)	40 (47.6) *	*0.619*	17 (48.6)	47 (49.5) *	*0.927*	71 (54.6)	26 (56.5%)	45 (53.6)	*0.735*	18 (51.4)	53 (55.8)	*0.731*
**T2DM (n,%)**	20/129 (15.5) *,#	9/45 (20.0) *	11 (13.1) *	*0.302*	7/34 (20.6) **	13 (13.7) *	*0.340*	33/129(25.5)	17/45 (37.0)	16 (19)	*0.025*	11/34 (31.4)	22 (23.2)	*0.337*
**Dyslipidemia (n,%)**	58 (44.6) *	17 (37)	41 (48.8) *	*0.174*	11 (31.4)	47 (49.4) *	*0.059*	72 (55.4)	21 (45.7)	51 (60.7)	*0.099*	15 (42.9)	57 (60.0)	*0.081*
**Obesity (n,%)**	57 (43.8)	19 (41.3)	38 (45.2)	*0.665*	14 (40%)	43 (45.3)	*0.591*	52 (40)	17 (37)	35 (41.7)	*0.599*	13 (37.1)	39 (41.1)	*0.686*
**CKD (n,%)**	5 (3.8) **	1 (2.2)	4 (4.8)	*0.463*	1 (2.9)	4/95 (4.2)	*0.722*	9 (6.9)	4 (8.7)	5 (6.0)	*0.556*	4 (11.4)	5 (5.3)	*0.219*
**Osteoporosis (n,%)**	23 (17.7) *	7 (15.2)	16 (19.0) *	*0.584*	4 (11.4)	19 (20.0) *	*0.256*	30 (23.1)	8 (17.4)	22 (26.2)	*0.255*	5 (14.3)	25 (26.3)	*0.149*
**Fractures (n,%)**	8 (6.2)	2 (4.3)	6 (7.1)	*0.526*	1 (2.9)	7 (7.4)	*0.342*	9 (6.9)	2 (4.3)	7 (8.3)	*0.518*	1 (2.9)	8 (8.4)	*0.442*
**Dementia (n,%)**	3 (2.3)	3 (6.5)	0 (0)	*0.018*	2 (5.7)	1 (1.1)	*0.116*	3 (2.3)	3 (6.5)	0 (0)	*0.018*	2 (5.7)	1 (1.1)	*0.116*
**Psychiatric diagnosis (n,%)**	19 (14.6) **	7 (15.2)	12 (14.3)	*0.886*	5 (14.3)	14 (14.7)	*0.949*	23 (17.7)	9 (19.6)	14 (16.7)	*0.679*	7 (20.0)	16 (16.8)	*0.676*
**CV thrombosis (n,%)**	8 (6.2)	2 (4.3)	6 (7.1)	*0.526*	2 (5.7)	6 (6.3)	*0.899*	9 (6.9)	3 (6.5)	6 (7.1)	*0.894*	3 (8.6)	6 (6.3)	*0.653*
**Myocardial infarction (n,%)**	5 (3.8)	3 (6.5)	2 (2.4)	*0.240*	2 (5.7)	3 (3.2)	*0.501*	5 (3.8)	3 (6.5)	2 (2.4)	*0.240*	2 (5.7)	3 (3.2)	*0.501*
**PTCA (n,%)**	3 (2.3)	1 (2.2)	2 (2.4)	*0.940*	1 (2.9)	2 (2.1)	*0.800*	4 (3.1)	1 (2.2)	3 (3.6)	*0.659*	1 (2.9)	3 (3.2)	*0.930*
**Heart failure**	1 (0.8)	1 (2.2)	0 (0)	*0.175*	1 (2.9)	0 (0)	*0.098*	2 (1.5)	2 (4.3)	0 (0)	*0.054*	2 (5.7)	0 (0)	***0.019***
**Atrial fibrillation (n,%)**	6 (4.6)	3 (6.5)	3 (3.6)	*0.443*	2 (5.7)	4 (4.2)	*0.717*	6 (4.6)	3 (6.5)	3 (3.6)	*0.443*	2 (5.7)	4 (4.2)	*0.717*
**Stroke (n,%)**	2 (1.5)	0 (0)	2 (2.4)	*0.292*	0 (0)	2 (2.1)	*0.387*	2 (1.5)	0 (0)	2 (2.4)	*0.292*	0 (0%)	2 (2.1)	*0.387*
**DVT (n,%)**	1 (0.8)	1 (2.2)	0 (0)	*0.175*	1 (2.9)	0 (0)	*0.098*	3 (2.3)	1 (2.2)	2 (2.4)	*0.940*	1 (2.9)	2 (2.1)	*0.800*
**Pulmonary embolism (n,%)**	0 (0)	0 (0)	0 (0)	*1.0*	0 (0)	0 (0)	*1.0*	1 (0.8)	0 (0)	1 (1.2)	*0.458*	0 (0)	1(1.1)	*0.542*

BMI: body-mass-index, F-ODS: cortisol after 1 mg dexamethasone suppression test, LLN: lower limit of normal, MACS: mild autonomous cortisol secretion, HTN: hypertension, T2DM: diabetes mellitus type 2, CKD: chronic kidney disease, CV: cardiovascular, PTCA: percutaneous transluminal coronary angioplasty, DVT: deep vein thrombosis, * *p* < 0.05 versus follow-up, ** *p* < 0.1 versus follow-up, and # one patient with type 1 diabetes mellitus.

**Table 3:** Demographics, hormonal data, and comorbidities of patients with NAPACA and the presence of MACS at the baseline visit and at the latest follow-up visit (>24 months).

	BASELINE			FOLLOW-UP		
	Non-MACS N = 97	MACS N = 33	*p*	Non-MACS N = 97	MACS N = 33	*p*
**ANY NEOPLASMS (n,%)**	38 (39.18)	8 (24.24)	*0.089*	Not applicable		
**MALIGNANCY (n,%)**	30 (30.93)	5 (15.15)	*0.058*	Not applicable		
**Age (years)**	59 (18–89)	65 (42–85)	*0.005*	63.5 (28–91)	70 (48–88)	*0.004*
**Male (n,%)**	18 (18.6)	10 (30.3)	*0.122*	Not applicable		
**BMI (kg/m^2^)**	29.09 (17.14–51.67)	28.16 (18.59–63.29)	*0.913*	29.26 (17.40–55.08)	28.58 (17.63–50.3)	*0.808*
**F-ODS (μg/dL)**	1.01 (0.3–1.9)	2.2 (1.8–4.33)	*<0.001*	1.01 (0.3–4.9)	2.4 (0.62–10.7)	*<0.001*
**ACTH (** **pg/mL)**	20.7 (5.6–200)	9.3 (3.8–36.4)	*<0.001*	20.2 (5–97.9)	12.2 (4.52–29.8)	*<0.001*
**DHEAS to LLN**	2.96 (0.51–13.0)	2.15 (0.51–10.2)	*0.013*	2.62 (0.34–13.0)	2.62 (0.51–12.3)	*0.098*
**BMI/F-ODS**	30.02 (13.88–120.17)	13.49 (6.27–28.77)	*<0.001*	28.26 (7.6–128.09)	11.37 (2.68–46.08)	*<0.001*
**ACTH/F-ODS**	19.29 (4–222.22)	4.38 (0.92–15.05)	*<0.001*	20 (3.05–163.25)	5 (0.5–48.35)	*<0.001*
**Tumor size (mm)**	8 (4.5–40.9) *	19 (7–42.0)	*<0.001*	8.05 (4.5–48.0)	19 (7–44.0)	*0.002*
**Delta size (mm)**	Not applicable			0 (−8 →25.5)	0 (−4 →7)	*0.931*
**Bilateral (n,%)**	26/96 (27.1)	18 (54.5)	*0.012*	33/96 (34.4)	18 (54.5)	*0.033*
**Smokers (n,%)**	34/93 (36.6)	18/32 (56.3)	*0.041*	Not assessed		
**Hypertension (n,%)**	41 (42.3) *	23 (69.7)	*0.009*	47 (48.5)	24 (72.7)	*0.036*
**T2DM (n,%)**	10/96 (10.4) *^,#^	10 (30.3)	*0.010*	22/96 (22.9)	11 (33.3)	*0.251*
**Dyslipidemia (n,%)**	36 (37.5) *	22 (66.7) *	*0.005*	45 (46.4)	27 (81.8)	*<0.001*
**Obesity (n,%)**	44 (45.4)	13 (39.4) **	*0.550*	43 (44.3)	9 (27.3)	*0.079*
**CKD (n,%)**	2 (2.1)	3 (9.1)	*0.103*	4 (4.1)	5 (15.2)	*0.046*
**Osteoporosis (n,%)**	15 (15.5)	8 (24.2)	*0.188*	18 (18.6)	12 (36.4)	*0.054*
**Fractures (n,%)**	6 (6.2)	2 (6.1)	*0.671*	6 (6.2)	3 (9.1)	*0.771*
**Dementia (n,%)**	3 (3.1)	0 (0)	*0.412*	3 (3.1)	0 (0)	*0.571*
**Psychiatric disorders (n,%)**	10 (10.3)	9 (27.3)	*0.022*	12 (12.4)	11 (33.3)	*0.015*
**CV thrombosis (n,%)**	3 (3.1)	5 (15.2)	*0.025*	3 (3.1)	6 (18.2)	*0.008*
**Myocardial infarction (n,%)**	2 (2.1)	3 (9.1)	*0.103*	2 (2.1)	3 (9.1)	*0.103*
**PTCA (n,%)**	0 (0)	3 (9.1)	*0.015*	0 (0)	4 (12.1)	*0.004*
**Heart failure (n,%)**	0 (0)	1 (3)	*0.254*	0 (0)	2 (6.1)	*0.063*
**Atrial fibrillation (n,%)**	1 (1)	5 (15.2)	*0.004*	1 (1)	4 (12.1)	*0.004*
**Stroke (n,%)**	0 (0)	2 (6.1)	*0.063*	0 (0)	2 (6.1)	*0.063*
**DVT (n,%)**	1 (1)	0 (0)	*1.000*	3 (3.1)	0 (0)	*0.571*
**Pulmonary embolism (n,%)**	0 (0)	0 (0)	N/A	0 (0)	1 (3)	*0.254*

BMI: body-mass-index, F-ODS: cortisol after 1 mg dexamethasone suppression test, LLN: lower limit of normal, MACS: mild autonomous cortisol secretion, T2DM: diabetes mellitus type 2, CKD: chronic kidney disease, CV: cardiovascular, PTCA: percutaneous transluminal coronary angioplasty, and DVT: deep vein thrombosis. * *p* < 0.05 versus follow-up, ** *p* < 0.1 versus follow-up, and ^#^ one patient with type 1 diabetes mellitus.

**Table 4:** Univariate and multivariate logistic regression analysis for the factors identified as significant for the development of a second neoplasm in NAPACA patients.

	Univariate Logistic Regression Analysis			Multivariate Logistic Regression Analysis		
	Odds Ratio	*p*	95% CI	Odds Ratio	*p*	95% CI
**Factors associated with the presence of any second neoplasms**						
**F-ODS baseline**	0.562	*0.046*	0.320–0.989	0.614	*0.112*	0.336–1.121
**DELTA SIZE**	0.842	*0.05*	0.708–1.000	0.849	*0.077*	0.708–1.018
**Factors associated with the presence of second malignant neoplasms**						
**F-ODS baseline**	0.468	*0.027*	0.239–0.916	0.528	*0.098*	0.248–1.125
**DELTA SIZE**	0.823	*0.051*	0.677–1001	0.826	*0.070*	0.672–1.016
**ACTH follow-up**	1.029	*0.025*	1.004–1.055	1.024	*0.099*	0.996–1.052

F-ODS: cortisol after 1 mg dexamethasone suppression test, ACTH: adrenocorticotropic hormone, CI: confidence interval.

## Data Availability

The raw data supporting the conclusions of this article will be made available by the authors on request.
